# Effect of empathy competence on moral sensitivity in Chinese student nurses: the mediating role of emotional intelligence

**DOI:** 10.1186/s12912-023-01650-w

**Published:** 2023-12-19

**Authors:** Fang Liu, Hengyu Zhou, Long Yuan, Ying Cai

**Affiliations:** Nursing School of Chongqing Medical University, No. 1 Medical College Road, Yuzhong District, Chongqing, China

**Keywords:** Empathy, Emotional Intelligence, Moral sensitivity, Student nurses

## Abstract

**Background:**

Ethical issues may pose challenges to nursing students entering clinical practice. Moral sensitivity can assist them in recognising existing moral situations and then taking adequate action. Identifying the variables associated with moral sensitivity may be useful in preparing to improve nursing students' moral sensitivity.

**Objectives:**

This study investigated empathy, emotional intelligence, and moral sensitivity in Chinese student nurses to explore the association among these three factors and to verify the mediating function of emotional intelligence in determining the connection between empathy and moral sensitivity.

**Design:**

This study used a cross-sectional correlational design.

**Setting and participants:**

Through convenience sampling, 239 fourth-year nursing undergraduates at a university in Western China were enrolled in this study.

**Methods:**

Nursing students who volunteered to participate in the study completed self-reported scales on empathy, emotional intelligence, and moral sensitivity between September and October 2022. The potential mediating effect was explored using the Process Macro and bootstrap method.

**Results:**

The nursing students' average scores were 39.62 ± 5.27 on moral sensitivity, 108.21 ± 15.49 on empathy, and 124.41 ± 13.66 on EI. Moral sensitivity was positively correlated with emotional intelligence (*r* = 0.454, *p* < 0.001) and empathy (*r* = 0.545, *p* < 0.001). Furthermore, empathy exerted a substantial direct effect on nursing students' moral sensitivity (*B* = 0.1424, *p* < 0.001). Emotional intelligence could mediate the indirect path from empathy to moral sensitivity. (*B* = 0.0372, *p* < 0.001).

**Conclusion:**

Emotional intelligence mediated the association between empathy and moral sensitivity. Thus, educational activities and programmes placing an emphasis on empathy and emotional intelligence may offer an alternative way to promote moral sensitivity in Chinese student nurses.

**Implications:**

Nursing educators can organise programmes to improve nursing students' emotional competence and professional values. Early exposure to clinical practice benefits nursing students a lot in terms of building interactions with patients and increasing emotional resonance. In addition, nursing educators should develop situational teaching in nursing ethics courses to help students cope with ethical issues.

## Introduction

 Nurses play an essential role in healthcare services and hold the responsibility to make the best decisions for patients. Rapid advances in medicine and diverse social and cultural values have increased the complexity of the healthcare system and caused high levels of ethical distress among nurses in their practice. The most common ethical issues include informed consent, equality of care, patients' privacy protection, and relationships with patients and other medical personnel [1]. Nurses are often stressed about ethical issues, which leads to professional burnout, increased turnover rates, decreased job satisfaction, and may even affect the quality of nursing services [2–4]. To resolve ethical issues, nurses must first be morally sensitive to identifying ethical problems. Moral sensitivity was defined as the ability to recognise a moral conflict, show a contextual and intuitive understanding of the patient's vulnerable situation, and comprehend the ethical consequences of the decisions made on behalf of the patient. It was a prerequisite for making ethical decisions and conducting moral behaviour [5]. Nurses with low moral sensitivity may not identify moral situations and react appropriately, which will cause unfavourable health outcomes and harm patients' rights.

Nursing student interns are undergoing the transition from student to nurse roles, and they must be able to recognize their own and others’ moral values. In the lower grades, they had limited opportunities to contact patients and experience specific issues in the hospital. Nursing students are exposed to morally challenging situations when they begin intensive clinical practice, and they require moral sensitivity to offer holistic care to patients based on reliable ethical decision-making skills [6]. Moral competences should be developed as a component of professional nursing. Previous research has indicated the existing level of moral sensitivity among nursing students is low, mainly due to the lack of relevant training during nursing education [7, 8]. Therefore, further researches on moral sensitivity are required to gain deep insights into its correlates, so as to improve it in an effective way.

## Background

Empathy and moral sensitivity are closely related, and both of them are vital for providing holistic nursing care. Empathy is often defined as the feeling of a person imagining himself in another's situation, which reflects the ability to understand how others feel and what it means, as well as to express these emotions to others [9]. Empathy is a fundamental element of nursing care that leads to better quality care and more harmonious nurse-patient relationships [10, 11]. Studies have noted that the level of moral sensitivity can be enhanced by recognizing empathy as one of the most important factors [12, 13]. Nurses with empathy competencies have the ability to understand patients' needs, concerns, and emotions, safeguard patients' rights and interests, and thus engage in rational moral behaviour in ethical dilemmas [14, 15]. Nursing students' empathy was revealed to have a positive effect on their ethical sensitivity, which could help them overcome ethical difficulties in the workplace [16]. However, empathy can sometimes interfere with moral behaviour by introducing partiality [17].

Emotional intelligence (EI) is the skill of understanding one's own and others' emotions and using them efficiently in various situations to help guide behaviour and thinking [18]. It has been proven that highly developed EI assists the individual in reducing mistakes and regulating emotions, and supports them in making more precise decisions [19]. Previous studies have explored the association between EI and moral sensitivity. Ye et al. [20] pointed out that EI positively predicted nurses' moral sensitivity. EI serves as a bridge between emotional and rational cognition, facilitating nurses to recognize and analyse emotional meanings and conduct cognitive reasoning with moral sensitivity. Nursing students' EI is equally important in the development of moral sensitivity. Nursing students with advanced EI will have a better understanding of emotions and how emotions affect ethical decision-making [21].

EI has also been demonstrated to have an important theoretical and empirical association with empathy among nursing students [22]. EI and empathy are a group of qualities that help people comprehend their own feelings as well as the feelings of others. In addition, EI involves a wide range of emotions and enables individuals to manage and use emotions to guide reasonable behaviours. The enhancement of EI is beneficial for the development of emotions and the formation of moderate empathic interactions by introducing rational thinking. Nursing students with higher EI are more empathetic and better at managing their emotions. Consequently, more emphasis should be placed on fostering the growth of these capabilities.

Despite the pairwise association among empathy, EI, and moral sensitivity that has been previously explored, the internal procedure by which the three variables affect each other remains unknown. This study aimed to explore the relationship between the three variables, and to identify the intermediate effect of EI on the relationship between empathy and moral sensitivity among student nurses. Furthermore, it targeted acquiring data for the construction of nursing programmes to promote the moral sensitivity of undergraduate nursing interns in China.

## Methods

### Study design

A cross-sectional correlational study was conducted to identify the mediating effect of EI on the association between empathy and moral sensitivity among student nurses.

### Setting and participants

Nursing programmes in China are composed of four years of study, including 10 months of internship. The first two years mainly involve the study of fundamental nursing knowledge and skills. In the third year, students begin the clinical nursing courses that allow them to go to the hospital for intermittent probation in limited times. The fourth (last) year comprises 10 months of clinical internship and is considered an intensive and compulsory training phase in which students must apply their academic knowledge and skills to real-world clinical care. Therefore, internships are regarded as an important transitional period for students becoming nurses.

Research subjects were chosen using the convenience sampling method among fourth-year nursing undergraduates at a university in Chongqing, China. The following were the inclusion criteria: (1) fourth-year nursing undergraduate interns, (2) internship ≥ 3 months in a tertiary hospital, (3) voluntarily participation in this study. Students who did not practice, suspended their practice, or refused to participate were excluded. A total of 251 students completed and returned questionnaires. However, 12 questionnaires were disqualified for regular responses throughout the questionnaire and thus were removed from the overall analysis. As a result, 239 students participated in the study, generating a 95.22% response rate.

### Measurements

#### Demographics

Participants' demographic characteristics included gender, age, residence, whether the student was from a single-child family, degree of preference for nursing, whether they participated in ethical knowledge training, and whether they were satisfied with their clinical practice.

#### Moral Sensitivity Questionnaire-Revised Version into Chinese (MSQ-R-CV)

The Moral Sensitivity Questionnaire was compiled by Lützén et al. [23] in 2001 and revised in 2006. In 2015, Huang et al. [1] translated this questionnaire into the Chinese version and adapt the original three dimensions into two dimensions: moral responsibility and strength (5 items), and sense of moral burden (4 items). It is a self-reported questionnaire that grades on a 6-point Likert scale (1 = strongly disagree, 6 = strongly agree) with a total score of 9–54. Higher total scores indicate greater ethical sensitivity. The Cronbach's α coefficient of the scale was 0.82.

#### Emotional Intelligence Scale-Version into Chinese (EIS-CV)

The EIS was designed by Schutte et al. [24] in 1998 and translated by Wang et al. [25] in 2002 to assess individual emotional intelligence. The scale consists of four dimensions: emotion perception (12 items), managing self- relevant emotions (8items), managing others’ emotions (6 items), and utilizing emotions (7 items), with 33 items in total. Some items (5,28,33) had inverted score. A Likert 5-point scale was used to assign 1–5 points from "very inconsistent" to "very consistent". The higher the total score, the higher the individual's emotional intelligence level. The Cronbach's α coefficient for the EIS-CV was 0.88.

#### Jefferson Scale of Physician Empathy-nursing student (JSPE-NS)

The scale was developed by Dr Hojat et al. [26] in 2002, and the Chinese scholars Qiu et al. [27] translated and revised it into Chinese version in 2010 to evaluate nursing students' empathy ability. It is a self-report questionnaire with 20 items divided into three dimensions: perspective taking (10 items), compassionate care (8 items), and standing in the patient's shoes (2 items). The scale was designed as a Likert7 scale (1 = totally disagree, 7 = totally agree), with a total score ranging from 20 to 140. The higher the total score, the better the nursing students' empathy. The scale's Cronbach's α coefficient was 0.836.

#### Data collection

Data collection was conducted through an online survey platform from September to October, 2022. Clinical practice teaching managers were the primary investigators who agreed to cooperate and receive the relevant training. Before the formal survey, a pre-survey with a sample size of 5% was conducted to ensure the clarity of the questionnaires. Following minor adjustments to the questionnaires, the formal survey was launched, and information was sent through online student work groups. Students were first given written explanations of the study's aims and process, principles of voluntary participation and anonymity. They had the option not to participate in or withdraw from the study, and these actions would not have any impact on their performance. Upon their agreement to participate and return of the signed electronic informed consent forms, participants were instructed to complete the questionnaire truthfully. It took approximately 15 min to fill out the entire questionnaire. All the collected data were encoded and used just for research purposes.

### Data analysis

Data analyses were performed using IBM SPSS version 26.0. The enumeration data were expressed as frequencies and composition ratios, and the measurement data were described as means and standard deviations. Differences in the moral sensitivity of students with various characteristics were compared using two independent sample t-tests and one-way ANOVA. Pearson's correlations were used to identify associations between variables. The mediating effect of EI was examined using Process Macro, and the bootstrap method was applied to determine the significance of the mediation effect. P ≤ 0.05 was regarded as statistically significant.

## Results

### Participants’ demographic characteristics and their distribution by moral sensitivity score

 The total of 239 participants consisted of 12.55% males and 87.45% females. The mean age was 21.21 ± 0.93, ranging from 18 to 24 years. 129 students (53.31%) lived in urban areas and 113 students (46.69%) lived in rural areas. The majority (69.87%) were not the only children in their family. Only 38.49% of the students liked nursing major, and 28.87% had participated in ethical knowledge training. In terms of satisfaction with the clinical practice, 80.33% of the participants reported contentment. There were no significant differences in moral sensitivity scores when participants were grouped according to gender, age, residence, family with only one child, or satisfaction with clinical practice. However, significant differences were found in some variables between the groups according to professional love and ethical knowledge training (Table 1).

**Table 1:** Participants' demographic characteristics and their distribution by moral sensitivity score (*N* = 239)

**Characteristics**	N (%)	**Moral Sensitivity**		
		Mean ± SD	*t*/*F*	P
**Gender**				
Male	30(12.55)	39.30 ± 5.58	0.354	0.724
Female	209(87.45)	39.67 ± 5.24		
**Age**				
19–20	43(17.99)	39.28 ± 4.82	0.186	0.831
21–22	179(74.90)	39.65 ± 5.35		
23–24	17(7.11)	40.18 ± 5.87		
**Residence**				
Urban	127(53.14)	39.71 ± 5.60	0.279	0.781
Rural	112(46.86)	39.52 ± 4.91		
**Family with only child**				
Yes	72(30.13)	39.86 ± 5.09	0.465	0.643
No	167(69.87)	39.51 ± 5.36		
**Professional love**				
Like	92(38.49)	40.79 ± 5.07	6.019	0.003
Moderate	135(56.49)	39.15 ± 5.24		
Dislike	12(5.02)	35.92 ± 5.02		
**Ethical knowledge training**				
Yes	69(28.87)	37.96 ± 4.72	3.163	0.002
No	170(71.13)	40.29 ± 5.35		
**Satisfaction with clinical practice**				
Yes	192(80.33)	39.80 ± 5.28	0.340	0.622
No	47(19.67)	39.66 ± 4.88		

### Mean scores and correlations among empathy, EI, and moral sensitivity

The nursing students' average scores were 39.62 ± 5.27 on moral sensitivity, 108.21 ± 15.49 on empathy and 124.41 ± 13.66 on EI. Correlations between empathy, EI and moral sensitivity revealed that moral sensitivity was positively associated with EI and empathy (*r* = 0.454, *p* < 0.001; *r* = 0.545, *p* < 0.001, respectively). Empathy was also positively associated with EI (*r* = 0.581, *p* < 0.001) (Table 2).

**Table 2:** Mean *scores* and correlations among the study variables (*N* = 239)

Variables	Mean ± SD	Moral Sensitivity	Emotional Intelligence
		**r**	**r**
**Moral Sensitivity**	39.62 ± 5.27	1	
**Emotional Intelligence**	124.41 ± 13.66	0.454^**^	
**Empathy**	108.21 ± 15.49	0.545^**^	0.581^**^

^**^P < 0.001

### Mediating effect of EI on the relationship between empathy and moral sensitivity

**Fig. 1:**
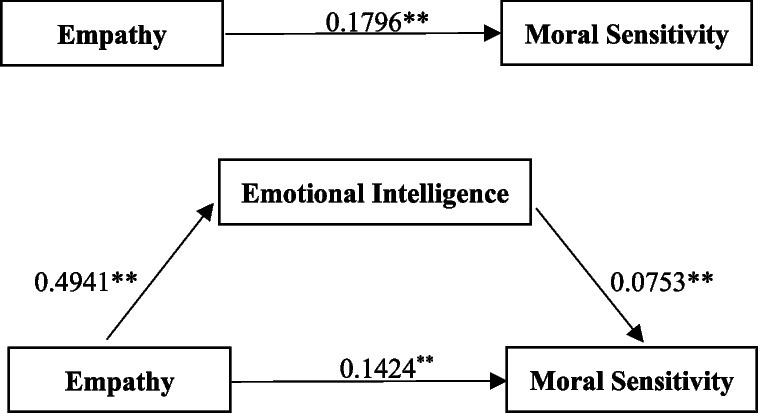
Mediation model of effects of emotional intelligence on the relationship between empathy and moral sensitivity (*N* = 239). **p < 0.001

 Regression analysis of the model's variables revealed that nursing students' empathy positively influenced their moral sensitivity (*B* = 0.1796, *p* < 0.001). Additionally, the degree of empathy had a direct impact on the students' EI (*B* = 0.4941, *p* < 0.001). When empathy and EI were simultaneously applied to predict moral sensitivity, both path coefficients were statistically significant (*B* = 0.1424, *p* < 0.001; *B* = 0.0753, *p* < 0.001) (Fig. 1).

The indirect effect size of the path (empathy → EI → moral sensitivity) was estimated to be 0.0372 as a result of the mediating influence on EI. The 95% confidence interval (CI) was 0.0067–0.0693, excluding 0, suggesting that the mediating effect of EI was significant. This finding demonstrated the existence of the intermediary path hypothesized in this study with a relative effect size of 20.71%. After introducing the mediating variable EI, the direct effect of empathy on moral sensitivity was 0.1424. The 95% CI was 0.0938–0.1928, excluding 0, indicating that empathy exerted a substantial direct effect on nursing students' moral sensitivity (Table 3).

**Table 3:** Mediating effects of emotional intelligence on the relationship between empathy and moral sensitivity (*N* = 239)

Effects	Effect size	SE	95% CI
**Total effect**			
Empathy → Moral Sensitivity	0.1796	0.018	0.149–0.221
**Direct effect**			
Empathy → Moral Sensitivity	0.1424	0.0255	0.0938–0.1928
**Indirect effect**			
Empathy → Emotional Intelligence → Moral Sensitivity	0.0372	0.0157	0.0067–0.0693

## Discussions

This study explored the relationship between empathy, EI, and moral sensitivity, and the mediating effect of EI on empathy and moral sensitivity among Chinese fourth-year nursing undergraduates. The results showed that empathy and EI were correlated with moral sensitivity, and EI acted as a mediator between empathy and moral sensitivity.

Empathy plays an important role in establishing effective therapeutics and helping relationships with patients [28]. The empathy score of nursing students in this study was similar to the findings of Su et al.[29] whose study involved second-year nursing students. Moreover, the score was lower than that reported in a previous investigation of fourth-year nursing students using the same tool [22]. These differences may be explained by the fact that senior nursing students focus more on improving their knowledge and skills in clinical practice, and lack empathy training. They cannot put themselves in the shoes of patients to perceive their emotional needs and provide emotional care.

EI is equally significant in influencing professional relationships and patient care decisions. The EI score in this study was lower than that reported in the prior two studies [21, 30]. This may be due to the fact that Chinese education pays little attention to the cultivation of students' EI at all stages, and students cannot adequately manage the challenges from their surroundings and cope with difficulties in their daily life.

Moral sensitivity implies the internalization of professional values that have become standards for behaviour and practice [31]. The results of this study indicated that professional love was one of the factors affecting nursing students' moral sensitivity. Students who preferred nursing were more sensitive to morality. If students cannot establish a professional identity, their professional values and attitudes are negative, and they tend to ignore common ethical issues in clinical practice and make unreasonable clinical decisions. The score of moral sensitivity in this study was at the medium level. Student nurses in this study presented a higher level of moral sensitivity than those found in a previous study involving second- and third-year nursing students [32]. This is attributed to the fact that Chinese nursing students in lower grades have fewer opportunities to encounter ethical issues, whereas nursing students in senior grades are more likely to experience ethical conflicts because of their entry into clinical practice. However, the moral sensitivity score of student nurses was lower than that of nurses [1]. Nurses have extensive work experience to deal with moral issues from the patient's perspective, and have sufficient courage to take actions and then justify their actions. This study revealed that the ethical knowledge training of student nurses was another important factor affecting their moral sensitivity. Therefore, nursing educators should increase ethical training and expand students' personal experiences in clinical practice, starting in the lower grades.

This study suggested that empathy had positive and direct effect on moral sensitivity of Chinese student nurses, which was in line with prior researches [13, 33]. Empathy is widely regarded as a crucial prerequisite for providing quality nursing care since it enables nurses to understand patients' demands, feelings, and situations. As an intrinsic element of the personality of medical personnel, empathy contributes to a vital function in improving interpersonal relationships and communication. Empathetic people usually have a profound understanding of other's emotions as well as a strong awareness of subtle issues. Moral sensitivity to moral issues in caring situations requires empathy competence to identify patients' feelings and sufferings and recognize moral values of the activities. Therefore, empathic competence can enhance moral sensitivity to moral affairs and help address moral issues related to ethical challenges among student nurses.

EI was also found to be a predictive factor of moral sensitivity among Chinese student nurses. That was consistent with previous research [20, 21]. Individuals with higher EI predict better emotion regulation ability, monitoring, and adjusting their psychology and behaviour, and they tend to be better at analysing and reasoning about moral concerns. When confronted with complex moral judgements or typical moral pressure, they usually make better moral decisions because they are aware of what is going on around them and can analyse issues more effectively, and then identify the right actions. Consequently, high moral sensitivity appears to be a positive consequence of high EI, suggesting that the EI of student nurses should be vigorously developed to promote moral sensitivity.

The findings showed that empathy exhibited a significant positive correlation with EI. Empathy and EI are both involved with the competency of emotions and feelings in humans. It can be declared that EI influences empathy by introducing emotional self-control. People with a higher level of EI possess stronger sympathetic skills as well as better capacities to regulate emotions and establish efficient coping strategies while dealing with ethical challenges.

To further identify the impact of empathy on students' moral sensitivity, this study analysed the mediation effects of EI and revealed the indirect effect of empathy on moral sensitivity through EI. Students with higher empathy scores may have better EI, thereby possessing higher moral sensitivity. EI is an important variable that can buffer the negative effect of excessive empathy on moral sensitivity, regulate personal emotion, and promote reasonable behaviour. These results are useful for a better understanding of the relationship between nursing students' empathy and moral sensitivity. Therefore, it has to be necessary to mediate EI along with empathy to strengthen the level of moral sensitivity.

### Implications

This study offers specific guidance for nursing education in developing paths to promote nursing students' moral sensitivity. It should be recognized that moral sensitivity can be fostered when combining empathy and EI together in the nursing curriculum. Nursing educators can organize programmes to increase nursing students' empathy and EI by introducing strategies for improving communication and sharing skills, understanding others' emotions and influencing them, promoting emotional self-management, and using emotions to guide rational behaviour. Professional value education is also necessary to develop the professional identity of nursing students so as to increase their moral sensitivity. Moreover, more clinical practice activities for nursing students should be carried out during the four years to build early interaction with patients, increase emotional resonance, and provide the opportunity for them to be exposed to the ethical context. In addition, nursing educators should attach importance to nursing ethics courses, diversify teaching methods, and conduct more situational simulations and role-playing to help them understand, analyse, and solve ethical issues.

### Limitations

This study holds certain limitations. Firstly, the cross-sectional study design restricted the ability to verify causal correlations among the variables. The hypotheses of this study should be further explored through longitudinal studies. Secondly, the participants were recruited using a convenience sample from only one nursing college, which may limit the generalisability of the conclusions to other groups. Thirdly, data collection relied primarily on self-reporting by nursing students, which may introduce the possibility of social desirability bias. Despite these limitations, this study provides insights into the definite relationship between empathy, EI, and moral sensitivity among Chinese student nurses, which to our knowledge has not been examined before.

## Conclusions

This study confirmed the role of EI as a mediator between moral sensitivity and empathy in student nurses, establishing an empirical foundation for strengthening the moral sensitivity of student nurses. In the future, we will concentrate on specialized intervention studies to explore efficient training strategies for effectively promoting emotional competency and moral sensitivity in Chinese nursing students.

## Data Availability

All data generated or analysed during this study are not publicly available due to the confidential agreement of participants, but are available from the corresponding author upon reasonable request.
